# Massive Pulmonary Embolism in a Recent Intracranial Hemorrhage: A Case Report of Inhaled Nitric Oxide to Improve Outcomes

**DOI:** 10.7759/cureus.8218

**Published:** 2020-05-21

**Authors:** Abhinav Mittal, Anne P Mittal, Edward Rojas, Hatim Al-Jaroushi

**Affiliations:** 1 Pulmonary, Critical Care & Sleep Medicine, West Virginia University, Morgantown, USA; 2 Neurocritical Care, West Virginia University, Morgantown, USA

**Keywords:** nitric oxide, massive pulmonary embolism, intracranial hemorrhage, inhaled nitric oxide, pulmonary vascular resistance, pulmonary arterial pressure, pulmonary hypertension

## Abstract

Acute massive pulmonary embolism (PE) has a high mortality if left untreated. The mainstay of treatment is systemic thrombolysis which has some absolute contraindications like intracranial hemorrhage (ICH). Inhaled nitric oxide (iNO) is a selective pulmonary vasodilator that decreases pulmonary artery pressure (PAP) and allows the right ventricle of the heart to pump against less resistance. We present a case of iNO use to improve hemodynamics in a patient with a recent ICH. We believe this to be the first such case reported.

A 70-year-old female with a history of PE on Eliquis initially presented for weakness and was found to have right-sided ICH. She was discharged with instructions to hold Eliquis given ICH but was readmitted eight days later in florid cardiogenic shock requiring vasopressors and hypoxic respiratory failure refractory to intubation. CT showed bilateral PE with evidence of right heart strain and IV heparin was started. Due to her history of a recent ICH, she had an absolute contraindication prohibiting the use of systemic tissue plasminogen activator (tPA). Interventional radiology (IR) consult determined that the patient was not a candidate for catheter-directed tPA due to the recent ICH, mechanical ventilation, and hemodynamic instability based on pressor requirement. Vascular surgery and extracorporeal membrane oxygenation (ECMO) consults deemed the patient not operable. The patient was then started on iNO with immediate improvement in her blood pressure. Once vitally stable, IR consult performed pulmonary angiogram and completed a thrombectomy. The patient was eventually extubated and she restarted her Eliquis. She continues to do well 16 months after discharge.

In patients with massive PE with contraindications to systemic thrombolytics, providers are left with very few therapeutic interventions. A handful of case reports show that iNO improves systemic hemodynamics in postoperative patients with massive PE. This case highlights the potential for iNO to be a potential adjuvant in patients with absolute contraindications to systemic thrombolysis.

## Introduction

Acute massive pulmonary embolism (PE) carries a >50% mortality in the first two hours of symptom onset [1]. Any embolus that occludes flow to the pulmonary vasculature rapidly increases distal pulmonary artery pressure (PAP) and pulmonary vascular resistance (PVR). Pulmonary circulation is typically a low-pressure system supported by a right ventricle (RV) that is normally only 1-3 mm thick. In acute PE, the sudden increase in afterload on an under-equipped RV leads to rapid dysfunction, which is directly correlated with patient outcomes [2]. While the standard of care to acutely improve RV afterload from massive PE is systemic thrombolytics, there are several contraindications limiting their use. Similarly, there is no consensus for the role of catheter directed thrombolysis (cdtPA) and even this approach has contraindications similar to those of systemic thrombolytics. Vasodilators like isoproterenol and nitrates have failed to have mortality benefit due to worsening of concurrent systemic hypotension.

Inhaled nitric oxide (iNO) is a selective pulmonary vasodilator that decreases PAP and PVR in primary pulmonary hypertension and is used off label in acute respiratory distress syndrome (ARDS), sickle cell crisis, and postcardiac surgery. It is short acting and causes vasodilation only in well-ventilated portions of the lung limiting ventilation/perfusion mismatch without causing systemic hypotension. Furthermore, the effect of iNO is achieved within minutes, and reversed promptly after discontinuation. There are a handful of case reports of using iNO as an adjuvant therapy to improve hemodynamics in patients with an acute massive PE in postsurgical patients prior to tissue plasminogen activator (tPA) [3-4]. We present a case report of iNO use to salvage hemodynamics in a patient with recent intracranial hemorrhage (ICH). With her improved hemodynamics, she was able to successfully undergo thrombectomy and survived. After review of current literature, we believe this to be the first such case reported.

## Case presentation

**Figure 1:**
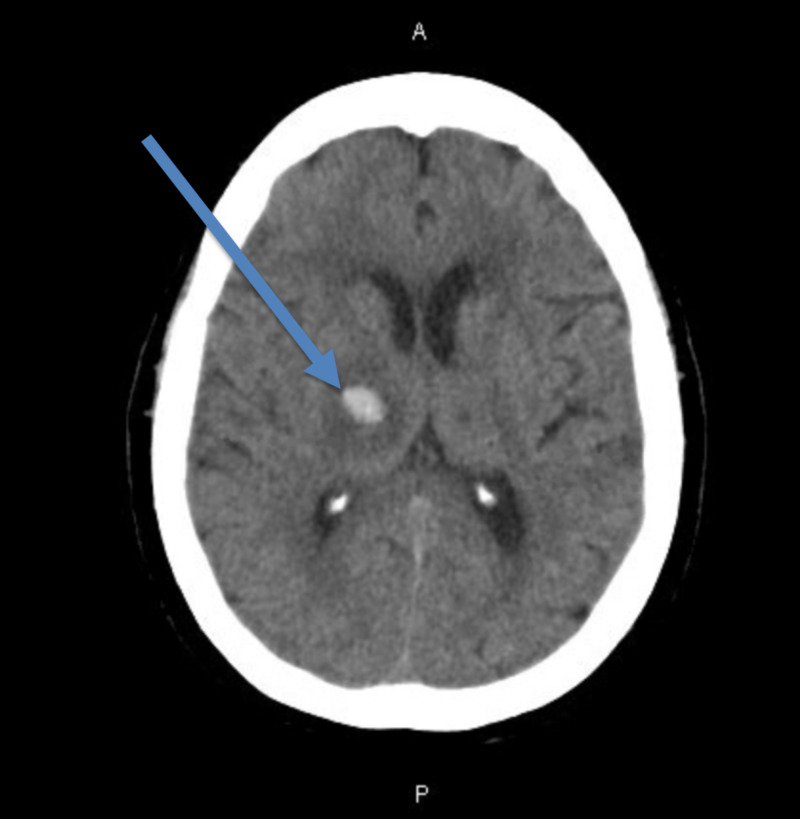
Axial image of CT of brain without intravenous contrast showing hyperdense right sided intra-cranial hemorrhage (blue arrow) that measures 13 mm x 14 mm with surrounding ring of hypodense cytotoxic edema. There is mild mass effect but no associated midline shift.

A 70-year-old female with a history of PE on Eliquis initially presented for left-sided weakness. Workup including intracranial CT angiography showed an acute right thalamic ICH (Figure 1).

**Figure 2:**
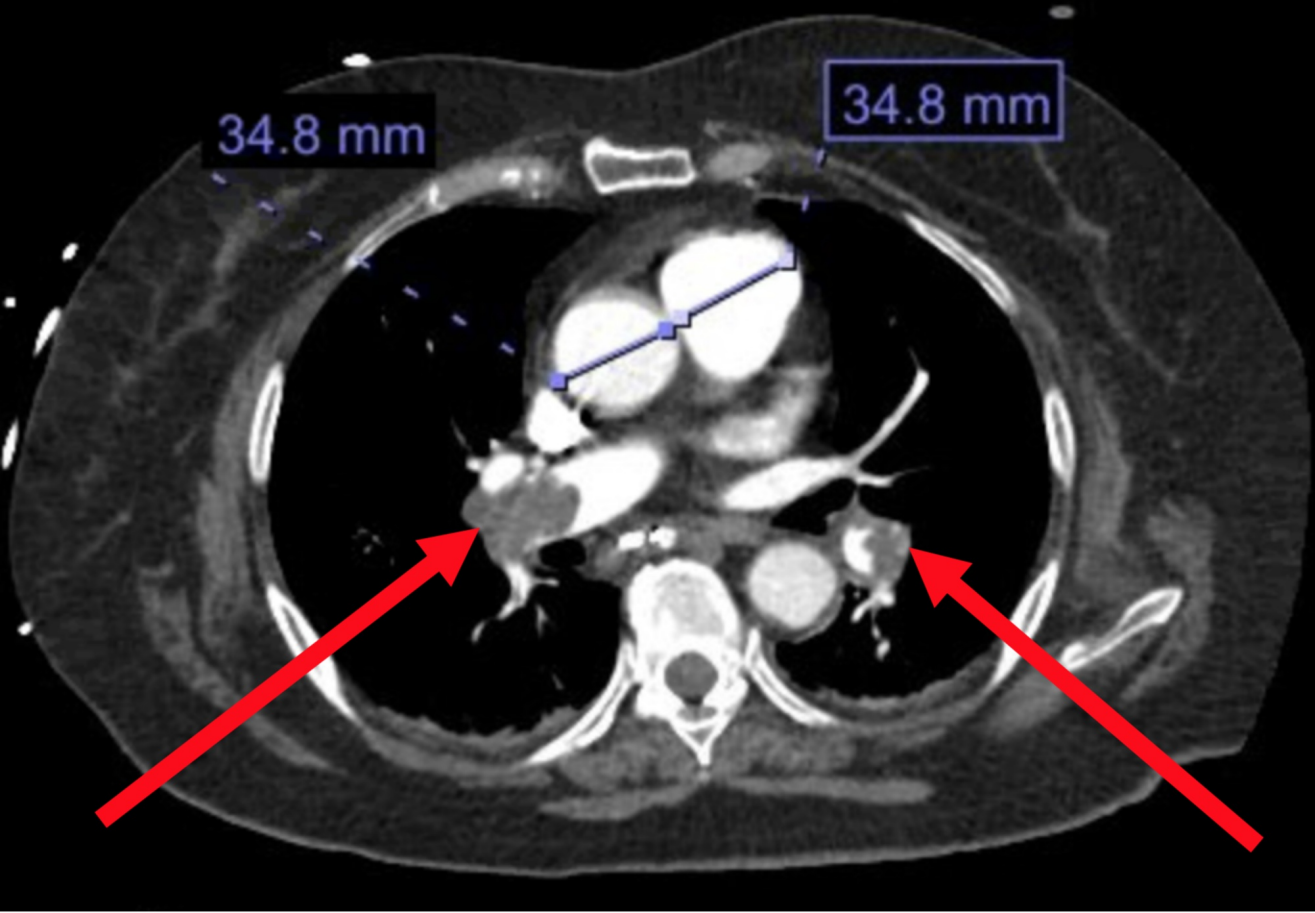
Axial image of CT of chest with intravenous contrast showing right main and left segmental pulmonary artery embolisms (red arrows). Measurements of the diameter of the pulmonary trunk and aorta are both 34.8 mm supporting radiographic evidence of massive pulmonary embolism.

Hypercoagulable workup was not revealing and decision was made to hold the Eliquis in the setting of her recent ICH. Unfortunately, the patient presented eight days later with shortness of breath and hypoxia. The patient was intubated but remained difficult to achieve adequate oxygenation and required vasopressor to maintain adequate blood pressure. CT PE study showed bilateral PE (Figure 2) with evidence of right heart strain. Furthermore, lab work demonstrated elevating troponins and basic natriuretic peptide indicating right heart strain. Intravenous heparin was started but patient's shock and hypoxia remained problematic to treat.

Given her recent ICH, she was not a candidate for systemic thrombolytics. Two separate interventional radiology (IR) attendings were consulted but determined that the patient was not a candidate for cdtPA due to recent ICH, mechanical ventilation, and relative vital instability. Vascular surgery and extracorporeal membrane oxygenation (ECMO) consults deemed that the patient was not an operative candidate given her do-not-resuscitate (DNR) status, co-morbidities, and poor functional baseline. The patient was then started on iNO at 40 parts per million with immediate improvement in her blood pressure and was successfully weaned off pressor support within 12 hours. Upon pressor discontinuation, IR completed a pulmonary angiogram showing elevated main PAP and completed suction mechanical thrombectomy with improvement in burden and PAP. Nitric oxide was weaned slowly off by hospital day 5 and she was extubated on hospital day 6. CT brain revealed resolution of her IPH and neurosurgery cleared the patient for resumption of Eliquis. The patient was discharged on day 8 and continues to do well at her follow up visit 16 months later.

## Discussion

Acute massive PE requires high index of clinical suspicion and prompt treatment. In patients who are not a candidate for acute surgical thrombectomy or thrombolytics, there is a relative void of treatment options at a provider’s disposal. iNO is an endogenous vasodilator that activates smooth muscles by increasing cyclic guanosine monophosphate (cGMP) levels. This rise in cGMP then activates protein kinase which dephosphorylates the light chains leading to arterial muscle relaxation [5].

The use of iNO has FDA indications for pulmonary hypertension for reducing PVR and PAP. Animal models with piglets showed that administration of iNO following massive PE selectively decreases PAP and PVR without affecting systemic hemodynamics [6]. The lack of systemic effect makes iNO more clinically applicable in situations of massive PE compared to other vasodilators like nitrates or isoproterenol. One case series showed iNO leads to improvement in PAP/PVR and also in systemic hemodynamics as a bridge to more definitive therapy in four postoperative patients [4]. Similarly, a case series of postabdominal surgery patients had similar systemic hemodynamic improvement with iNO [3].

This case highlights the potential for iNO to be a potential bridge therapy in acute massive PE in patients who may not be candidates for thrombolytic therapy, as in this patient with an ICH one week prior to presentation. iNO may help improve hemodynamics enough to receive definitive therapies in this population, which could potentially be lifesaving. Further studies need to be completed to address this possibility.

## Conclusions

In our review of the literature, this is the first case demonstrating the utility of iNO as a bridge for massive PE in a patient with a recent ICH. While iNO has been reported in postsurgical patients, this case highlights the utility of iNO to stabilize hemodynamics in a rapidly deteriorating nonsurgical patient. This novel use of iNO allowed the patient to undergo definitive thrombectomy and afforded her a positive outcome. Future studies are needed to establish the role of iNO as an adjuvant in the management of massive PE. 
